# Dissecting Cellulitis Presenting After Hair Restoration Surgery

**DOI:** 10.7759/cureus.74304

**Published:** 2024-11-23

**Authors:** Guillermo A Guerrero-González, Gerardo González-Martínez, Jair A Valdez-Zertuche

**Affiliations:** 1 Dermatologic Surgery, Pango Dermatología, Monterrey, MEX; 2 Dermatology, Hospital Universitario “Dr. José Eleuterio González”, Nuevo León, MEX; 3 Dermatology, Hospital Universitario "Dr. José Eleuterio González", Nuevo León, MEX

**Keywords:** 5-alpha reductase inhibitors, dissecting cellulitis, fue hair transplant, hair transplantation, scarring alopecia

## Abstract

Dissecting cellulitis (DC) of the scalp is a chronic inflammatory condition marked by neutrophilic cicatricial alopecia, often linked to staphylococcal antigens. This case report details a 34-year-old male with scarring acne who developed DC following follicular unit extraction (FUE) approximately four months prior. Trichoscopic examination revealed brown pigmented dots, erythema, and melicerous crusts. Abscess drainage yielded negative bacterial cultures, leading to a diagnosis of DC. The patient was treated with isotretinoin (20 mg/day), dutasteride (0.5 mg/day), intralesional steroid injections, and salicylic acid-based shampoo. After four months, the patient exhibited significant improvement, with trichoscopic findings showing regrowing hairs and no abnormal hair loss. DC, though commonly associated with other follicular occlusion disorders, has not been previously reported following FUE, indicating a need for awareness of this potential complication. The efficacy of isotretinoin and the emerging role of dutasteride in managing DC highlight the importance of early diagnosis and personalized treatment. Trichoscopy is crucial for diagnosis and monitoring, emphasizing the need for prompt intervention to prevent scarring alopecia. Caution is advised when considering FUE in patients with severe inflammatory acne due to the risk of developing DC.

## Introduction

Dissecting cellulitis (DC) of the scalp is a chronic inflammatory disorder characterized by neutrophilic cicatricial alopecia, often associated with staphylococcal antigenic triggers [1]. It presents most commonly in men with a dark phototype and is more common in patients with other follicular occlusion disorders (hidradenitis suppurativa, acne conglobata, and pilonidal cyst). Clinical findings include nodules and abscesses in the vertex region, though other areas may be affected. When left untreated, the abnormal inflammatory response can cause complications such as scarring alopecia, lymphadenopathy, and, in rare cases, squamous cell carcinoma [2]. Histopathological examination of DC lesions reveals significant inflammatory infiltrates in the hair follicle’s infundibulum and isthmus, leading to a foreign body reaction and subsequent fibrosis replacing hair follicle structures. The precise mechanisms driving follicular destruction in DC remain unclear [1,3]. Differential diagnoses include acne keloidalis nuchae and folliculitis decalvans. Although there is no standard treatment to date, common options include oral antibiotics and systemic retinoids, with recent publications using biologic drugs [4].

This case report details a patient with DC following follicular unit extraction (FUE), emphasizing the importance of early diagnosis and individualized treatment to prevent irreversible sequelae.

## Case presentation

**Figure 1:**
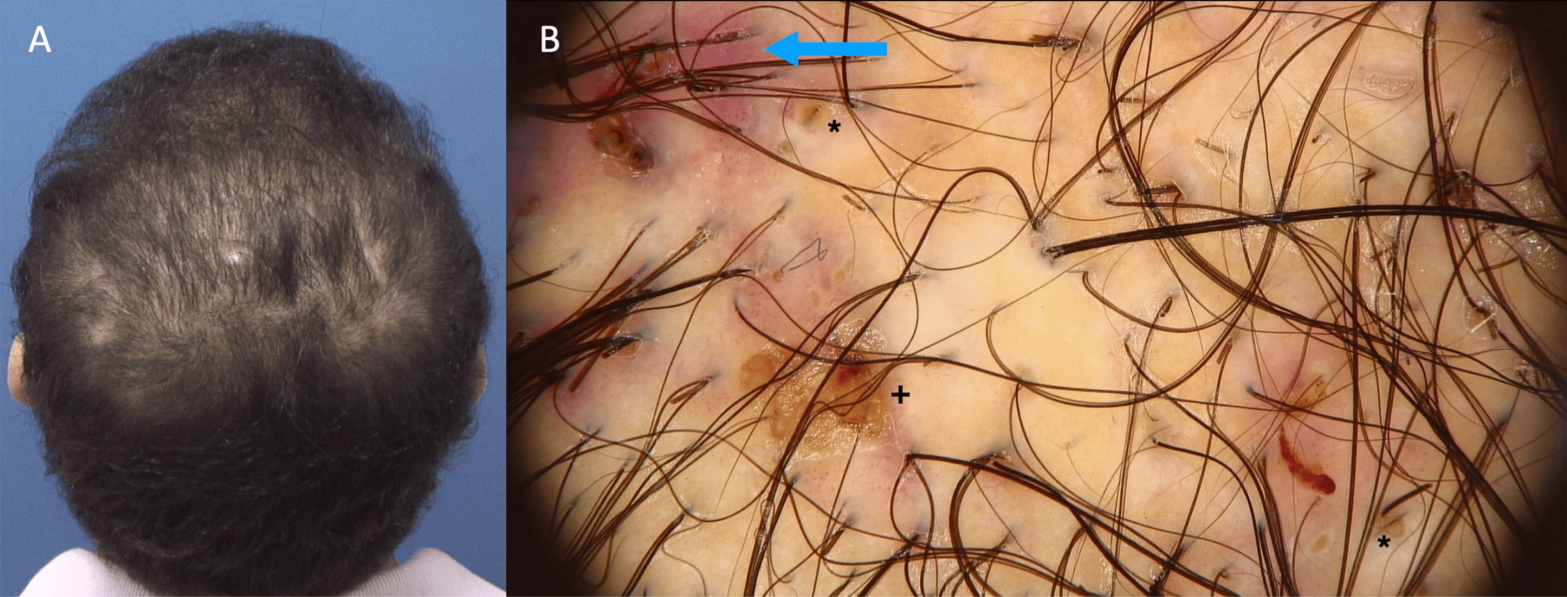
Patient four months after undergoing FUE A) Patient with multiple fluctuating scalp lesions and alopecic patches in the occipital region. B) Trichoscopy showing large brown dots (*) and peri-and interfollicular erythema (arrow) and crusting (+). FUE: follicular unit extraction

A 34-year-old male with a history of androgenetic alopecia (AGA) and acne presented with multiple fluctuating scalp lesions four months after undergoing FUE (Figure 1A). Trichoscopy revealed large brown pigmented dots, interfollicular and perifollicular erythema, and melicerous crusts, indicative of inflammation (Figure 1B). Abscess drainage was performed, with cultures yielding negative bacterial growth. Based on these findings, a diagnosis of DC was established.

**Figure 2:**
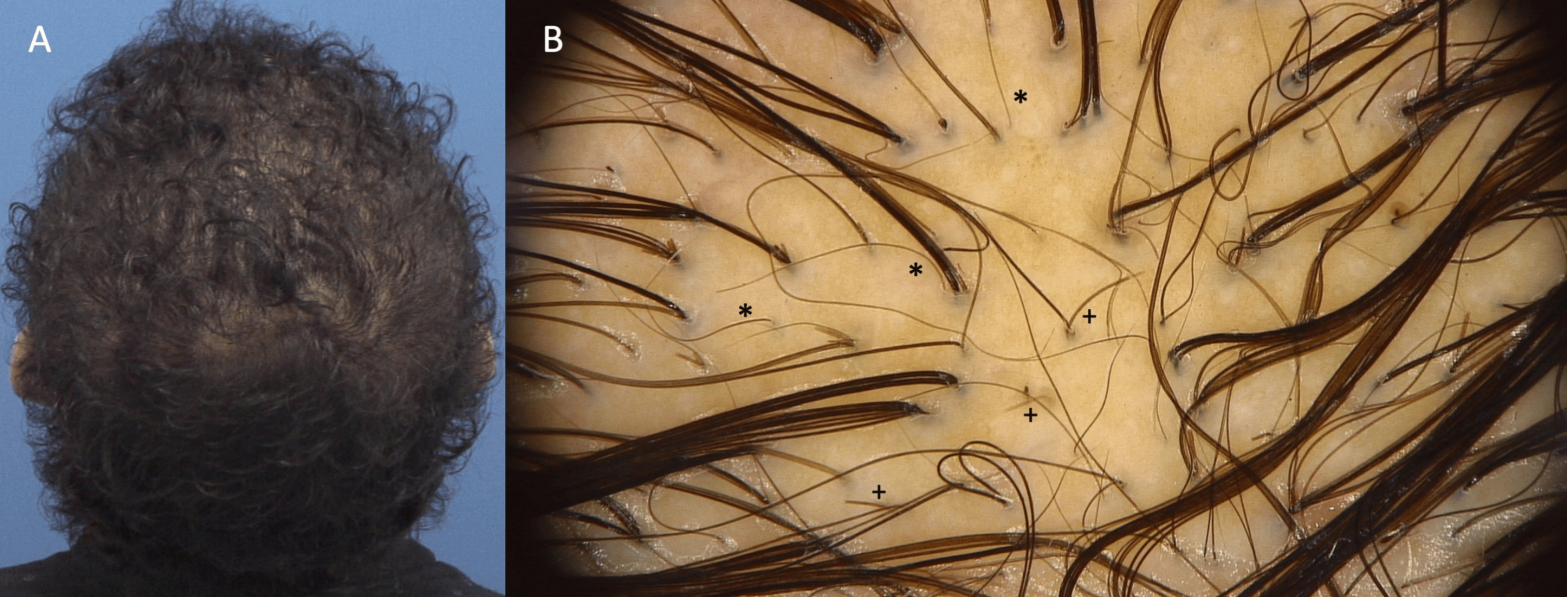
The patient four months after treatment A) Patient with an almost complete response. B) Trichoscopy revealing no evidence of abnormal or absent hair growth but showing downy (*) and regrowing hairs (+).

The patient was treated with isotretinoin (20 mg/day), dutasteride (0.5 mg/day), intralesional steroid injections (two sessions, four weeks apart), and daily salicylic acid-based shampoo. After four months, the patient showed an almost complete response, with trichoscopy revealing downy and regrowing hairs and no abnormal hair loss (Figures 2A, 2B).

## Discussion

DC is commonly seen in patients with follicular occlusion disorders, such as acne conglobata, hidradenitis suppurativa, and pilonidal cysts, but it has not been reported following FUE, making its incidence unclear as a post-procedure complication [5,6]. Trichoscopy is valuable in diagnosing DC, often revealing findings like 3D yellow dots, black dots, and exclamation mark hairs [7], which align with our observations in this case. Untreated DC can lead to scarring alopecia, highlighting the need for prompt treatment.

Despite there being no standard treatment for this condition due to its low incidence, several studies have demonstrated the effectiveness of oral isotretinoin in managing patients with DC [8-10]. Notably, a meta-analysis reported an estimated overall efficacy rate of isotretinoin in treating DC of the scalp of 0.9, with a 95% confidence interval of 0.81 to 0.97. Sensitivity analysis further supported the robustness of this efficacy, with a range from 0.83 to 0.94 [11].

In recent years, dutasteride has gained recognition as a therapeutic agent for AGA, demonstrating both high efficacy and tolerability [12,13] but also demonstrating a dual effect in reducing concomitant inflammatory acne [14]. Furthermore, as outlined in our case presentation, the use of dutasteride resulted in a favorable response in a patient presenting DC following hair transplantation with a history of AGA and acne. These findings suggest that dutasteride may play a crucial role in attenuating the risk of this rare complication.

## Conclusions

Given the rarity of DC, a standardized therapeutic approach is lacking. However, the therapeutic potential of dutasteride, particularly in AGA, is noteworthy, offering a promising intervention for reducing the incidence of DC post-hair transplantation. Trichoscopic findings play a crucial role in facilitating the diagnosis of DC. By leveraging these findings and gaining a comprehensive understanding of the natural course of the disease, physicians can make informed decisions and initiate timely therapy. It is imperative to emphasize its high propensity to progress into a scarred area if left untreated, underscoring the importance of prompt intervention to prevent long-term sequelae. Moreover, patients with a history of severe inflammatory acne should be carefully evaluated before undergoing FUE due to the increased risk of developing complications such as DC.
